# (*R*_p_)-2-Isopropyl-5-methyl­cyclo­hexyl isoprop­yl(phen­yl)phosphinate

**DOI:** 10.1107/S1600536811013390

**Published:** 2011-04-22

**Authors:** Li-Juan Liu, Hao Xu, Fan-Jie Meng, Da-Qi Wang, Chang-Qiu Zhao

**Affiliations:** a College of Chemistry and Chemical Engineering, Liaocheng University, Shandong 252059, People’s Republic of China

## Abstract

The title compound, C_19_H_31_O_2_P, features a distorted tetra­hedral P atom that bonds to the phenyl ring, isopropyl and 2-isopropyl-5-methyl­cyclo­hexyl groups, and is determined as having an *R*_p_ configuration. A chair conformation is observed for the cyclo­hexyl ring. In the crystal, mol­ecules are linked into chains running along the *a* axis by weak inter­molecular C—H⋯O hodrogen bonds.

## Related literature

For general background to P-chiral compounds and for related structures, see: Chaloner *et al.* (1991 ▶); Fu & Zhao *et al.* (2010 ▶).
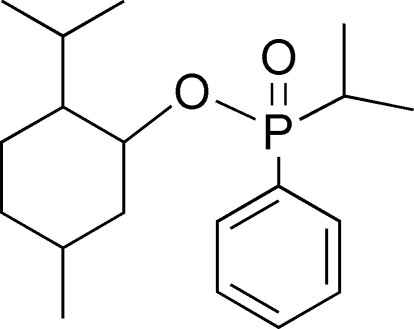


## Experimental

### 

#### Crystal data

C_19_H_31_O_2_P
*M* *_r_* = 322.41
Monoclinic, 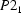

*a* = 5.8847 (4) Å
*b* = 17.196 (3) Å
*c* = 9.7075 (9) Å
β = 95.184 (1)°
*V* = 978.3 (2) Å^3^
*Z* = 2
Mo *K*α radiation
μ = 0.15 mm^−1^
*T* = 298 K
0.35 × 0.16 × 0.14 mm

#### Data collection

Siemens SMART 1000 CCD area-detector diffractometer
Absorption correction: multi-scan (*SADABS*; Sheldrick, 1996 ▶) *T*_min_ = 0.951, *T*_max_ = 0.980
4964 measured reflections
3175 independent reflections
1991 reflections with *I* > 2σ(*I*)
*R*_int_ = 0.045

#### Refinement

*R*[*F* ^2^ > 2σ(*F* ^2^)] = 0.047
*wR*(*F* ^2^) = 0.073
*S* = 1.00
3175 reflections
205 parameters
1 restraint
H-atom parameters constrained
Δρ_max_ = 0.22 e Å^−3^
Δρ_min_ = −0.16 e Å^−3^
Absolute structure: Flack (1983 ▶), 1775 Friedel pairs
Flack parameter: 0.02 (11)

### 

Data collection: *SMART* (Siemens, 1996 ▶); cell refinement: *SAINT* (Siemens, 1996 ▶); data reduction: *SAINT*; program(s) used to solve structure: *SHELXTL* (Sheldrick, 2008 ▶); program(s) used to refine structure: *SHELXTL*; molecular graphics: *SHELXTL*; software used to prepare material for publication: *SHELXTL*.

## Supplementary Material

Crystal structure: contains datablocks I, global. DOI: 10.1107/S1600536811013390/xu5176sup1.cif

Structure factors: contains datablocks I. DOI: 10.1107/S1600536811013390/xu5176Isup2.hkl

Additional supplementary materials:  crystallographic information; 3D view; checkCIF report

## Figures and Tables

**Table 1:** Hydrogen-bond geometry (Å, °)

*D*—H⋯*A*	*D*—H	H⋯*A*	*D*⋯*A*	*D*—H⋯*A*
C17—H17⋯O2^i^	0.98	2.44	3.180 (4)	132

Symmetry code: (i) 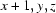.

**Fig. 1.**
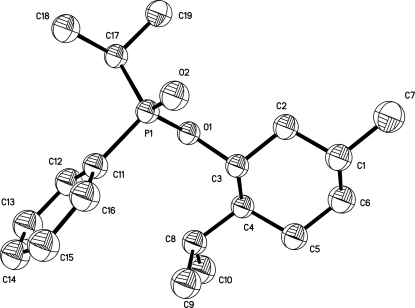
The molecular structure of the title compound, showing the atomic numbering scheme with 30% probability displacement ellipsoids.

**Fig. 2.**
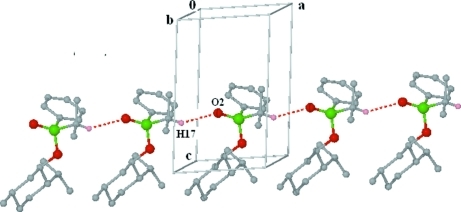
A view of the one-dimensional chain structure formed by C—H···O hodrogen bonds in the title compound. H atoms not involved in hydrogen bondings have been omitted for clarity. [Symmetry codes: (i) x + 1, y, z]

## supplementary crystallographic information

### Comment

The P-chiral compound has been reported previously (Chaloner *et al.*, 1991). We recently reported the crystal stucture of (*R*_p_)-α-hydroxy-cyclohexyl-menthyl phenylphosphinate, a compound readily synthesized by addition of (*R*_p_)-phenylphosphinate to cyclohexanone (Fu & Zhao, 2010). Herein we report a similar compound which is obtained by reaction of *O*-menthyl phenylphosphoryl chloride and isopropyl magnesium chloride.

A stable chair conformation is observed for the cyclohexane ring of the 2-isopropyl-5-methylcyclohexyloxy, in which the isopropyl, methyl and oxygen atom locate at equatorial bond. The absolute configuration of C_1_, C_3_, and C_4_ are *R*, *R*, and *S*, respectively (Fig.1). In this P-chiral title compound, the configuration of the central P atom is *R* and four groups around the P atom form an irregular tetrahedron. The bond angle of C11—P1—C17 is 107.61 (17)°, O1—P1—C11 is 105.66 (14)°, O1—P1—C17 is 101.39 (13)°, O2—P1—O1 is 115.03 (12)°, O2—P1—C17 is 114.76 (15)° and O2—P1—C11 is 111.50 (17)° (Chaloner *et al.* 1991). In the crystal structure, intermolecular C17—H17···O2 hodrogen bonds connect molecules into a one-dimensional chain (Fig.2).

### Experimental

*O*-Menthyl phenylphosphoryl chloride (0.3 mmol) was added to a stirred ether solution of isopropyl magnesium chloride (0.6 mmol) in a Schlenk tube under nitrogen, and the mixture was stirred for 24 h at room temperature. After washing with water, the resulting solution was purified by silica gel plate to afford the title compound. The crystal suit for X-ray diffraction was obtained from recrystallization with ethyl ether/hexane.

### Refinement

H atoms were placed geometrically and treated as riding with C—H = 0.93 - 0.98 Å, with *U*_iso_(H) = 1.5*U*_eq_(C) for methyl H and *U*_iso_(H) = 1.2*U*_eq_(C) for all other H atoms.

### Crystal data

**Table:**

C_19_H_31_O_2_P	*F*(000) = 352
*M**_r_* = 322.41	*D*_x_ = 1.094 Mg m^−^^3^
Monoclinic, *P*2_1_	Mo *K*α radiation, λ = 0.71073 Å
Hall symbol: P 2yb	Cell parameters from 1152 reflections
*a* = 5.8847 (4) Å	θ = 3.2–18.6°
*b* = 17.196 (3) Å	µ = 0.15 mm^−^^1^
*c* = 9.7075 (9) Å	*T* = 298 K
β = 95.184 (1)°	Block, colorless
*V* = 978.3 (2) Å^3^	0.35 × 0.16 × 0.14 mm
*Z* = 2	

### Data collection

**Table:**

Siemens SMART 1000 CCD area-detector diffractometer	3175 independent reflections
Radiation source: fine-focus sealed tube	1991 reflections with *I* > 2σ(*I*)
graphite	*R*_int_ = 0.045
φ and ω scans	θ_max_ = 25.0°, θ_min_ = 2.4°
Absorption correction: multi-scan (*SADABS*; Sheldrick, 1996)	*h* = −6→7
*T*_min_ = 0.951, *T*_max_ = 0.980	*k* = −20→20
4964 measured reflections	*l* = −9→11

### Refinement

**Table:**

Refinement on *F*^2^	Secondary atom site location: difference Fourier map
Least-squares matrix: full	Hydrogen site location: inferred from neighbouring sites
*R*[*F*^2^ > 2σ(*F*^2^)] = 0.047	H-atom parameters constrained
*wR*(*F*^2^) = 0.073	*w* = 1/[σ^2^(*F*_o_^2^) + (0.0093*P*)^2^] where *P* = (*F*_o_^2^ + 2*F*_c_^2^)/3
*S* = 1.00	(Δ/σ)_max_ = 0.001
3175 reflections	Δρ_max_ = 0.22 e Å^−^^3^
205 parameters	Δρ_min_ = −0.16 e Å^−^^3^
1 restraint	Absolute structure: Flack (1983), 1775 Friedel pairs
Primary atom site location: structure-invariant direct methods	Flack parameter: 0.02 (11)

### Special details

**Table:**

Geometry. All e.s.d.'s (except the e.s.d. in the dihedral angle between two l.s. planes) are estimated using the full covariance matrix. The cell e.s.d.'s are taken into account individually in the estimation of e.s.d.'s in distances, angles and torsion angles; correlations between e.s.d.'s in cell parameters are only used when they are defined by crystal symmetry. An approximate (isotropic) treatment of cell e.s.d.'s is used for estimating e.s.d.'s involving l.s. planes.
Refinement. Refinement of *F*^2^ against ALL reflections. The weighted *R*-factor *wR* and goodness of fit *S* are based on *F*^2^, conventional *R*-factors *R* are based on *F*, with *F* set to zero for negative *F*^2^. The threshold expression of *F*^2^ > σ(*F*^2^) is used only for calculating *R*-factors(gt) *etc*. and is not relevant to the choice of reflections for refinement. *R*-factors based on *F*^2^ are statistically about twice as large as those based on *F*, and *R*- factors based on ALL data will be even larger.

### Fractional atomic coordinates and isotropic or equivalent isotropic displacement parameters (Å^2^)

**Table:**

	*x*	*y*	*z*	*U*_iso_*/*U*_eq_	
P1	0.58068 (16)	0.52850 (6)	0.70438 (9)	0.0580 (2)	
O1	0.6203 (3)	0.51752 (13)	0.86663 (18)	0.0574 (6)	
O2	0.3626 (4)	0.56419 (13)	0.6548 (2)	0.0764 (8)	
C1	0.1496 (7)	0.5651 (2)	1.0974 (3)	0.0740 (11)	
H1	0.0193	0.5439	1.0393	0.089*	
C2	0.3498 (6)	0.57668 (19)	1.0062 (3)	0.0657 (10)	
H2A	0.3025	0.6119	0.9310	0.079*	
H2B	0.4772	0.6003	1.0612	0.079*	
C3	0.4266 (5)	0.50056 (18)	0.9469 (3)	0.0565 (10)	
H3	0.3015	0.4784	0.8857	0.068*	
C4	0.5012 (6)	0.4420 (2)	1.0600 (3)	0.0633 (10)	
H4	0.6252	0.4662	1.1192	0.076*	
C5	0.3002 (7)	0.4311 (2)	1.1490 (4)	0.0801 (12)	
H5A	0.1737	0.4076	1.0927	0.096*	
H5B	0.3456	0.3954	1.2239	0.096*	
C6	0.2205 (7)	0.5062 (2)	1.2093 (4)	0.0858 (13)	
H6A	0.3427	0.5279	1.2714	0.103*	
H6B	0.0923	0.4957	1.2626	0.103*	
C7	0.0780 (7)	0.6421 (2)	1.1575 (4)	0.1057 (15)	
H7A	0.2041	0.6638	1.2145	0.159*	
H7B	−0.0475	0.6336	1.2124	0.159*	
H7C	0.0323	0.6775	1.0837	0.159*	
C8	0.5943 (7)	0.3651 (2)	1.0072 (4)	0.0768 (12)	
H8	0.7117	0.3787	0.9459	0.092*	
C9	0.4161 (8)	0.3168 (2)	0.9222 (4)	0.1041 (15)	
H9A	0.4885	0.2731	0.8831	0.156*	
H9B	0.3433	0.3482	0.8492	0.156*	
H9C	0.3039	0.2988	0.9806	0.156*	
C10	0.7115 (7)	0.3175 (2)	1.1254 (4)	0.1071 (15)	
H10A	0.5986	0.2963	1.1802	0.161*	
H10B	0.8137	0.3502	1.1820	0.161*	
H10C	0.7960	0.2758	1.0883	0.161*	
C11	0.6101 (7)	0.4337 (2)	0.6323 (3)	0.0630 (10)	
C12	0.8067 (8)	0.3905 (2)	0.6580 (4)	0.0767 (12)	
H12	0.9302	0.4118	0.7116	0.092*	
C13	0.8240 (9)	0.3154 (3)	0.6050 (4)	0.0929 (13)	
H13	0.9577	0.2870	0.6235	0.112*	
C14	0.6443 (10)	0.2842 (3)	0.5264 (5)	0.0956 (15)	
H14	0.6557	0.2344	0.4900	0.115*	
C15	0.4474 (9)	0.3255 (3)	0.5005 (4)	0.0925 (14)	
H15	0.3240	0.3035	0.4478	0.111*	
C16	0.4299 (7)	0.4003 (2)	0.5524 (4)	0.0777 (12)	
H16	0.2955	0.4282	0.5331	0.093*	
C17	0.8282 (6)	0.58385 (19)	0.6703 (3)	0.0597 (9)	
H17	0.9645	0.5550	0.7063	0.072*	
C18	0.8349 (7)	0.5941 (2)	0.5134 (3)	0.0848 (12)	
H18A	0.7075	0.6252	0.4776	0.127*	
H18B	0.8271	0.5441	0.4694	0.127*	
H18C	0.9744	0.6195	0.4952	0.127*	
C19	0.8278 (7)	0.66248 (18)	0.7434 (4)	0.0817 (13)	
H19A	0.9603	0.6916	0.7238	0.123*	
H19B	0.8300	0.6545	0.8414	0.123*	
H19C	0.6928	0.6908	0.7111	0.123*	

### Atomic displacement parameters (Å^2^)

**Table:**

	*U*^11^	*U*^22^	*U*^33^	*U*^12^	*U*^13^	*U*^23^
P1	0.0455 (6)	0.0754 (6)	0.0541 (5)	0.0036 (6)	0.0104 (4)	0.0064 (5)
O1	0.0461 (14)	0.0737 (16)	0.0541 (13)	−0.0016 (13)	0.0140 (10)	0.0055 (12)
O2	0.0450 (16)	0.113 (2)	0.0721 (15)	0.0172 (13)	0.0087 (12)	0.0088 (13)
C1	0.075 (3)	0.090 (3)	0.059 (2)	0.007 (2)	0.021 (2)	0.001 (2)
C2	0.067 (3)	0.073 (3)	0.059 (2)	−0.001 (2)	0.0167 (19)	0.0001 (19)
C3	0.052 (2)	0.071 (3)	0.049 (2)	−0.0088 (19)	0.0133 (18)	0.0024 (18)
C4	0.067 (3)	0.069 (3)	0.056 (2)	−0.004 (2)	0.015 (2)	0.005 (2)
C5	0.084 (3)	0.087 (3)	0.073 (3)	0.000 (2)	0.027 (2)	0.018 (2)
C6	0.090 (3)	0.106 (4)	0.066 (3)	0.000 (3)	0.033 (2)	0.002 (2)
C7	0.123 (4)	0.109 (4)	0.091 (3)	0.030 (3)	0.044 (3)	−0.012 (2)
C8	0.081 (3)	0.075 (3)	0.079 (3)	0.006 (2)	0.029 (2)	0.021 (2)
C9	0.126 (4)	0.084 (3)	0.104 (4)	−0.005 (3)	0.019 (3)	−0.006 (3)
C10	0.111 (4)	0.101 (4)	0.112 (4)	0.014 (3)	0.029 (3)	0.030 (3)
C11	0.057 (3)	0.081 (3)	0.052 (2)	−0.004 (2)	0.012 (2)	0.004 (2)
C12	0.064 (3)	0.082 (3)	0.085 (3)	−0.001 (2)	0.013 (2)	−0.007 (2)
C13	0.094 (4)	0.090 (4)	0.097 (4)	0.010 (3)	0.025 (3)	0.001 (3)
C14	0.116 (5)	0.087 (4)	0.087 (4)	−0.004 (4)	0.026 (3)	−0.015 (3)
C15	0.104 (5)	0.106 (4)	0.069 (3)	−0.029 (3)	0.009 (3)	−0.014 (3)
C16	0.073 (3)	0.098 (4)	0.062 (3)	−0.007 (3)	0.008 (2)	−0.003 (2)
C17	0.049 (2)	0.072 (3)	0.060 (2)	0.0058 (19)	0.0132 (17)	0.0110 (19)
C18	0.087 (3)	0.099 (3)	0.072 (3)	−0.009 (3)	0.028 (2)	0.020 (2)
C19	0.090 (3)	0.068 (3)	0.088 (3)	−0.008 (2)	0.012 (2)	0.009 (2)

### Geometric parameters (Å, °)

**Table:**

P1—O2	1.464 (2)	C8—H8	0.9800
P1—O1	1.5826 (19)	C9—H9A	0.9600
P1—C11	1.789 (4)	C9—H9B	0.9600
P1—C17	1.795 (3)	C9—H9C	0.9600
O1—C3	1.467 (3)	C10—H10A	0.9600
C1—C6	1.515 (4)	C10—H10B	0.9600
C1—C7	1.522 (4)	C10—H10C	0.9600
C1—C2	1.549 (4)	C11—C12	1.378 (5)
C1—H1	0.9800	C11—C16	1.381 (4)
C2—C3	1.515 (4)	C12—C13	1.398 (5)
C2—H2A	0.9700	C12—H12	0.9300
C2—H2B	0.9700	C13—C14	1.358 (6)
C3—C4	1.525 (4)	C13—H13	0.9300
C3—H3	0.9800	C14—C15	1.363 (6)
C4—C8	1.537 (5)	C14—H14	0.9300
C4—C5	1.538 (4)	C15—C16	1.388 (5)
C4—H4	0.9800	C15—H15	0.9300
C5—C6	1.511 (4)	C16—H16	0.9300
C5—H5A	0.9700	C17—C19	1.527 (4)
C5—H5B	0.9700	C17—C18	1.537 (4)
C6—H6A	0.9700	C17—H17	0.9800
C6—H6B	0.9700	C18—H18A	0.9600
C7—H7A	0.9600	C18—H18B	0.9600
C7—H7B	0.9600	C18—H18C	0.9600
C7—H7C	0.9600	C19—H19A	0.9600
C8—C9	1.521 (5)	C19—H19B	0.9600
C8—C10	1.523 (5)	C19—H19C	0.9600
			
O2—P1—O1	115.03 (12)	C9—C8—H8	106.8
O2—P1—C11	111.50 (17)	C10—C8—H8	106.8
O1—P1—C11	105.66 (14)	C4—C8—H8	106.8
O2—P1—C17	114.76 (15)	C8—C9—H9A	109.5
O1—P1—C17	101.39 (13)	C8—C9—H9B	109.5
C11—P1—C17	107.61 (17)	H9A—C9—H9B	109.5
C3—O1—P1	120.03 (18)	C8—C9—H9C	109.5
C6—C1—C7	112.0 (3)	H9A—C9—H9C	109.5
C6—C1—C2	108.7 (3)	H9B—C9—H9C	109.5
C7—C1—C2	111.0 (3)	C8—C10—H10A	109.5
C6—C1—H1	108.3	C8—C10—H10B	109.5
C7—C1—H1	108.3	H10A—C10—H10B	109.5
C2—C1—H1	108.3	C8—C10—H10C	109.5
C3—C2—C1	112.0 (3)	H10A—C10—H10C	109.5
C3—C2—H2A	109.2	H10B—C10—H10C	109.5
C1—C2—H2A	109.2	C12—C11—C16	117.9 (4)
C3—C2—H2B	109.2	C12—C11—P1	121.9 (3)
C1—C2—H2B	109.2	C16—C11—P1	120.2 (3)
H2A—C2—H2B	107.9	C11—C12—C13	121.3 (4)
O1—C3—C2	107.6 (2)	C11—C12—H12	119.4
O1—C3—C4	109.1 (3)	C13—C12—H12	119.4
C2—C3—C4	111.9 (3)	C14—C13—C12	119.6 (4)
O1—C3—H3	109.4	C14—C13—H13	120.2
C2—C3—H3	109.4	C12—C13—H13	120.2
C4—C3—H3	109.4	C13—C14—C15	120.2 (5)
C3—C4—C8	114.6 (3)	C13—C14—H14	119.9
C3—C4—C5	107.4 (3)	C15—C14—H14	119.9
C8—C4—C5	113.4 (3)	C14—C15—C16	120.4 (5)
C3—C4—H4	107.0	C14—C15—H15	119.8
C8—C4—H4	107.0	C16—C15—H15	119.8
C5—C4—H4	107.0	C11—C16—C15	120.7 (4)
C6—C5—C4	113.2 (3)	C11—C16—H16	119.6
C6—C5—H5A	108.9	C15—C16—H16	119.6
C4—C5—H5A	108.9	C19—C17—C18	111.1 (3)
C6—C5—H5B	108.9	C19—C17—P1	110.4 (2)
C4—C5—H5B	108.9	C18—C17—P1	109.7 (2)
H5A—C5—H5B	107.7	C19—C17—H17	108.5
C5—C6—C1	111.6 (3)	C18—C17—H17	108.5
C5—C6—H6A	109.3	P1—C17—H17	108.5
C1—C6—H6A	109.3	C17—C18—H18A	109.5
C5—C6—H6B	109.3	C17—C18—H18B	109.5
C1—C6—H6B	109.3	H18A—C18—H18B	109.5
H6A—C6—H6B	108.0	C17—C18—H18C	109.5
C1—C7—H7A	109.5	H18A—C18—H18C	109.5
C1—C7—H7B	109.5	H18B—C18—H18C	109.5
H7A—C7—H7B	109.5	C17—C19—H19A	109.5
C1—C7—H7C	109.5	C17—C19—H19B	109.5
H7A—C7—H7C	109.5	H19A—C19—H19B	109.5
H7B—C7—H7C	109.5	C17—C19—H19C	109.5
C9—C8—C10	111.0 (3)	H19A—C19—H19C	109.5
C9—C8—C4	113.7 (3)	H19B—C19—H19C	109.5
C10—C8—C4	111.3 (3)		
			
O2—P1—O1—C3	33.5 (3)	C5—C4—C8—C10	−68.5 (4)
C11—P1—O1—C3	−89.9 (3)	O2—P1—C11—C12	176.4 (3)
C17—P1—O1—C3	157.9 (2)	O1—P1—C11—C12	−58.0 (3)
C6—C1—C2—C3	55.5 (4)	C17—P1—C11—C12	49.7 (3)
C7—C1—C2—C3	179.1 (3)	O2—P1—C11—C16	−6.1 (3)
P1—O1—C3—C2	−97.1 (3)	O1—P1—C11—C16	119.6 (3)
P1—O1—C3—C4	141.3 (2)	C17—P1—C11—C16	−132.7 (3)
C1—C2—C3—O1	−178.1 (3)	C16—C11—C12—C13	0.0 (5)
C1—C2—C3—C4	−58.3 (4)	P1—C11—C12—C13	177.6 (3)
O1—C3—C4—C8	−57.7 (4)	C11—C12—C13—C14	0.2 (6)
C2—C3—C4—C8	−176.6 (3)	C12—C13—C14—C15	−0.7 (7)
O1—C3—C4—C5	175.3 (3)	C13—C14—C15—C16	1.1 (7)
C2—C3—C4—C5	56.4 (4)	C12—C11—C16—C15	0.3 (6)
C3—C4—C5—C6	−56.6 (4)	P1—C11—C16—C15	−177.4 (3)
C8—C4—C5—C6	175.8 (3)	C14—C15—C16—C11	−0.8 (6)
C4—C5—C6—C1	57.9 (5)	O2—P1—C17—C19	61.8 (3)
C7—C1—C6—C5	−177.8 (4)	O1—P1—C17—C19	−62.8 (3)
C2—C1—C6—C5	−54.7 (4)	C11—P1—C17—C19	−173.5 (2)
C3—C4—C8—C9	−66.2 (4)	O2—P1—C17—C18	−61.0 (3)
C5—C4—C8—C9	57.7 (4)	O1—P1—C17—C18	174.4 (2)
C3—C4—C8—C10	167.7 (3)	C11—P1—C17—C18	63.7 (3)

### Hydrogen-bond geometry (Å, °)

**Table:**

*D*—H···*A*	*D*—H	H···*A*	*D*···*A*	*D*—H···*A*
C17—H17···O2^i^	0.98	2.44	3.180 (4)	132

Symmetry codes: (i) *x*+1, *y*, *z*.
